# nn-TransUNet: An Automatic Deep Learning Pipeline for Heart MRI Segmentation

**DOI:** 10.3390/life12101570

**Published:** 2022-10-09

**Authors:** Li Zhao, Dongming Zhou, Xin Jin, Weina Zhu

**Affiliations:** 1 School of Information Science and Engineering, Yunnan University, Kunming 650504, China; 2 School of Software, Yunnan University, Kunming 650504, China

**Keywords:** medical imaging, MRI segmentation, convolutional neural network, vision transformer

## Abstract

Cardiovascular disease (CVD) is a disease with high mortality in modern times. The segmentation task for MRI to extract the related organs for CVD is essential for diagnosis. Currently, a large number of deep learning methods are designed for medical image segmentation tasks. However, the design of segmentation algorithms tends to have more focus on deepening the network architectures and tuning the parameters and hyperparameters manually, which not only leads to a high time and effort consumption, but also causes the problem that the architectures and setting designed for a single task only performs well in a single dataset, but have low performance in other cases. In this paper, nn-TransUNet, an automatic deep learning pipeline for MRI segmentation of the heart is proposed to combine the experiment planning of nnU-net and the network architecture of TransUNet. nn-TransUNet uses vision transformers and convolution layers in the design of the encoder and takes up convolution layers as decoder. With the adaptive preprocessing and network training plan generated by the proposed automatic experiment planning pipeline, nn-TransUNet is able to fulfill the target of medical image segmentation in heart MRI tasks. nn-TransUNet achieved state-of-the-art level in heart MRI segmentation task on Automatic Cardiac Diagnosis Challenge (ACDC) Dataset. It also saves the effort and time to manually tune the parameters and hyperparameters, which can reduce the burden on researchers.

## 1. Introduction

Cardiovascular disease (CVD) is the causation of a large number of deaths in modern medical domain [1]. The number of mortality caused by CVD in 2030 is estimated to be 23.6 million people per year [2]. Nowadays, with the spread of covid-19, it is observed that the cases of acute cardiac injury related to covid-19 is increasing rapidly [3], which gives the diagnosis of CVD more importance. In recent years, deep learning-based algorithms have been implemented for medical images processing and assisting diagnosis [4,5,6,7,8]. Medical image segmentation is a necessary step in the MRI based diagnosis. The task of MRI segmentation is to classify the raw medical images with specialized labels to highlight the organs that are needed for diagnosis in order to make more accurate and convincing diagnosis. In the process of image segmentation, the properties of medical image, such as shades of gray, colour, texture, brightness, contrast are used for segmenting and labelling the regions for each image. For deep learning-based CVD diagnosis methods, segmentation is a key part of preprocessing. The quality of the preprocessing task will have a direct influence on the predictions of the neural network models, which makes heart MRI segmentation even more important.

Currently, convolutional neural network (CNN) architectures with encoder-decoder structures such as FCN [9] and U-net [10] over-perform other network architectures and play the most important role in image segmentation tasks. The development of image segmentation algorithms based on CNN architectures has been intensive in recent years. The appearance of Fully Convolutional Network (FCN), a segmentation algorithm based on CNN proposed by Long et al. [9], is a milestone for image segmentation. FCN segments source images at pixel level (i.e., classify each pixel of input image). It solves the problem of image segmentation at the semantic level and is the start point of the encoder-decoder neural network architectures for image segmentation. Based on the study of FCN, U-net is proposed by Ronneberger et al. [10] in the same year. Compared to its baseline algorithm FCN, U-net improves the performance on the segmentation task and the training process is also more efficient. The influence of U-net is greater than that of FCN, especially the design concept of its U-shaped network architecture. U-net and the network architectures derived from U-net still prevail in the domain of image segmentation today.

Derived from U-net, several U-shape architectures were presented such as V-net [11], U-net++ [12], Y-net [13], M-net [14]. These architectures increase the segmentation performance by slightly changing the shape or deepening the network architectures of the U-net. However, the manual tuning process for model training with deep network architecture is time and computational resource consuming. datasets for the image processing tasks are much larger than the those for other tasks. A single training case for image processing always consists of multiple dimensions: position indices and RGB values, which makes the dataset massive. If a manual tuning process is to be implemented, the researcher has to wait for a long time for the training process to be completed, which is expensive. Another potential problem is that the hand-tuned setups are likely to perform well well only on a dedicated dataset, rather than on all scenarios and multiple datasets, which makes the results and performance less convincing.

In image processing, the consumption of computational resources such as GPU RAM is unavoidable as the requirements of batch size and patch size in model training increase. To achieve high performance in the segmentation task, the training process is also always time-consuming. However the manual hyperparameter-tuning process may cost additional time and the GPU utlization does not reach the limit if the batch size and patch size are manually tuned, which increases the cost of network training. To solve this problem, Isensee et al. [15] proposed nnU-net, an automatic experiment planning frame for image segmentation tasks. It uses a generic U-net as the network architecture and generates training plan based on the dataset properties and the device performance, and automatically executes the training process without manual tuning. nnU-net has been shown to perform well on multiple datasets.

Transformer is a deep learning architecture proposed by Vaswani et al. [16] initially for Nature Language Processing (NLP) tasks. Nowadays, transformer has gradually become popular in the image processing domain, and is implemented to image segmentation tasks [17]. The advantage of creating visual representations from image sequences makes Vision Transformer (VIT) [18] an alternative solution to image segmentation tasks other than CNN-based architectures. But in the computer vision domain, VIT is limited by the drawback that its performance of leveraging spatial context is not as good as CNNs [19]. To avoid this limitation, Chen et al. [20] proposed TransUNet using VITs as encoder and a CNN architecture as decoder. TransUNet achieves state-of-the-art performance on the Synapse multi-organ segmentation dataset, showing the potential of VIT-based architectures overperform CNNs in image segmentation tasks.

The aim of this study is to develop an automatic deep learning algorithm the task of cardiac MRI that can boost the clinic diagnosis accuracy of CVD and avoid performance degradation caused by manual parameter tuning. Thus, nn-TransUNet, an automatic deep learning pipeline for heart MRI segmentation is proposed combining TransUNet [20] and nnU-net [15]. TransUNet is a U-shape network architecture with Vision-Transformers (VIT) [18] for medical image segmentation, while nnUNet is a training plan generative pipeline using a generic U-net architecture for a wide range of image segmentation tasks. When the dataset is fed into the pipeline, nn-TransUNet will first generate a plan for pre-processing and schedule based on the properties of the source image, and then automatically run preprocessing. After preprocessing, the network topology is adapted according to the image size. The training process will eventually be done automatically.

The sections of the paper are organized as follows: Section 2 provides an introduction and background knowledge of related works; a discussion of current segmentation algorithms will also be given. The definition and implementation of the nn-TransUNet pipeline will be given in Section 3. Section 4 consists of the implementation details, experimental results and evaluation. Finally, the limitations of nn-TransUnet and future works will be discussed and conclusions will be reached.

## 2. Related Works

In this section, the related methods (i.e., nnU-net and TransUnet) will be introduced and discussed in detail.

Since proposed by Isensee et al. [15] in 2020, nnU-net has achieved state-of-the-art level in several domains of medical image segmentation. It even ranked first in 19 international deep learning image segmentation competitions. nnU-net consists of sufficient knowledge in the deep learning domain, including the properties of MRIs, the preprocessing techniques and common setting used for network training, while using a generic U-net as the network architecture.

**Figure 1:**
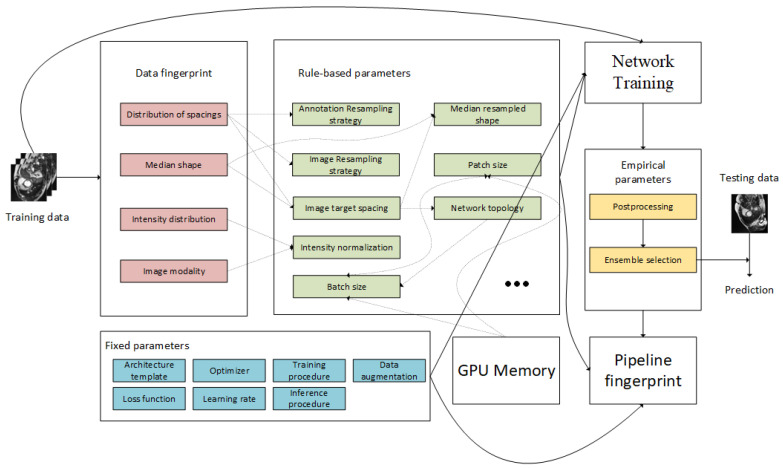
nnU-net automated method configuration [15].

As shown in Figure 1, when the dataset is input into nnU-net frame, the data properties are first calculated, which is, data fingerprint (highlighted in pink). Then the strategy and setting for dataset preprocessing and network training are generated according to data fingerprint and is regarded as the rule-based parameters (green). The network training settings in rule-based parameters are also related to the GPU memory limit, which means that the combination of batch size and patch size should not exceed the RAM of the GPU. The fixed parameters, marked in blue, are used for network training. The fixed parameters are the settings commonly used in the training process of deep learning models. A 5-fold cross-validation is performed to find the checkpoint of the model with the best performance and to address the potential missingness of the training case. After training and post-processing, ensemble selection can be used for prediction. The experiment plan, along with the saved weights checkpoint of the model, is saved as a pipeline fingerprint, which can be accessed and used for prediction and evaluation.

As the name suggests, nnU-net (no new U-net) is not a new network architecture. It is an automatic image segmentation training plan generative pipeline based on U-net, which means the architecture for nnU-net is a generic U-net [10].

**Figure 2:**
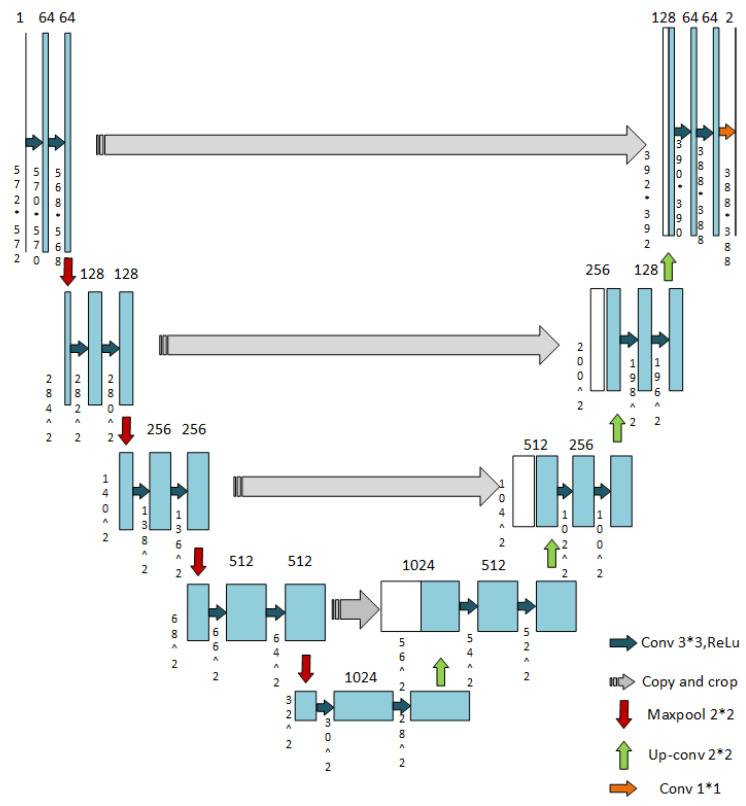
U-net architecture [10].

As shown in Figure 2, the network architecture of the generic U-net is a typical encoder-decoder CNN architecture. The encoder of the U-net, is also named as contraction path which performs convolution and down-sampling operations, while the decoder, also known as expanding path, up-samples the compressed representation at the bottleneck of the encoder to the input resolution and outputs the prediction. The corresponding layers of the path are connected by a skip-connection operation, which copies and concatenates the output of contracted path to the feature map of the expanding path in order to retain the low-resolution information that could potentially be lost when down-sampling the input image. Each layer of both contraction path and expanding path consist of 2 convolution layers of 3 × 3 with a Rectified Linear Unit (ReLU) activation function, the difference between the paths is that after convolution operations, the contraction path performs a down-sampling operation using a max pooling layer of 2 × 2, which cut the output to half while the expanding path used an up-convolution layer of 2 × 2, which doubles the output after convolution layers. In the last layer of expanding path, a 1 × 1 convolution layer is used to reduce the channels while keeping the size of the output. After the layer, the segmentation output is finally generated.

In 2021, Chen et al. [20] proposed TransUNet, a new network architecture for medical image segmentation algorithm using vision transformer as encoder. TransUNet also uses the skip-connection techniques, which makes TransUNet a U-shaped architecture. TransUNet uses a vision transformer as an encoder to compress the input image into a low-dimensional representation and uses a U-net-like expanding path with skip-connection for up-sampling. It achieves state-of-the-art performance in multi-organs MRI segmentation tasks.

The key concept of TransUNet is the vision transformer. Vision transformer [18] is a new approach of image segmentation, which is a combination of knowledge from both NLP (Natural Language Processing) and CV (Computer Vision) domain. It divides the input image into blocks, then flattens them into a sequence, inputs it into the encoder of the transformer, and finally outputs a segmentation map via a fully connected layer.

A vision transformer contains a multi-head self-attention block and a multi-layer perceptron block. Attention mechanism [21] is a technique which is broadly implemented in neural network architectures. It highlights the important parts of the input sequence by giving them higher weights. Multi-head self-attention blocks perform attention operations with multiple outputs to learn multi-modal features [22]. The architecture of a multi-layer perceptron is a finite acyclic graph consisting of logistic activation nodes [23]. Multi-layer perceptrons enable the neural networks to learn multi-level features from the data to solve complex problems.

For the implementation of the transformer, the input images need to be sequentialized and embedded accordingly. An input image is first sequentialized. The input image *x* is flattened to 2-D patches $\left\{x_{p}^{i}\in R^{P^{2}\cdot C}|i=1,\ldots ,N\right\}$, the size of each patch is P×P and *N* is the number of patches. Then a trainable linear projection is used to map the patches $x_{p}$ into a latent D-dimensional representation. The positive embeddings are learned and added to the patch embeddings to retain the location information. The process is shown in the equation below:

(1) $$z_{0}=\left[x_{p}^{1}E;x_{p}^{2},\ldots ,x_{p}^{N}\right]+E_{pos}$$

where *E* stands for the patch embedding projection while $E_{pos}$ is the embbeding position.

**Figure 3:**
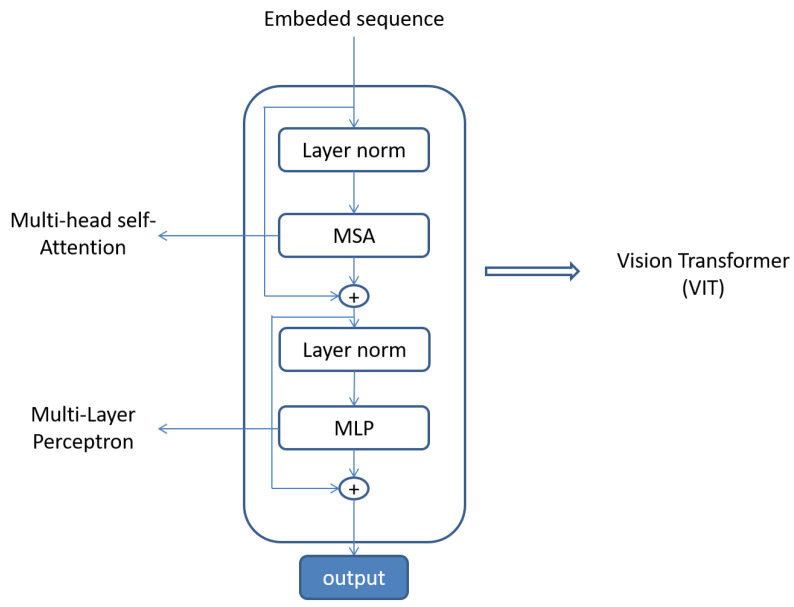
Transformer layer [20].

The encoder of TransUNet contains multiple transformer layers. The number of transformer layers *L* is 12 by default. A transformer layer consists of a multi-head self-attention (MSA) block and multi-layer perceptron (MLP) block. The output of the *n*-th transformer layer is shown below:

(2) $$z_{n}^{{}^{\prime }}=MSA\left(LN\left(z_{n-1}\right)\right)+Z_{n-1}$$

(3) $$z_{n}=MLP\left(LN\left(z_{n}^{{}^{\prime }}\right)\right)+z_{n}^{{}^{\prime }}$$

where LN is the representation of layer normalization operation. The architecture of a single transformer layer is shown in Figure 3.

## 3. Method

Based on nnU-net and TransUNet, nn-TransUNet proposes an automatic deep learning pipeline for heart MRI segmentation. While nn-TransUNet uses the same set of heuristic rules that are used for nnU-net to generate preprocessing and training plans for network training, TransUNet is the network architecture for nn-TransUNet.

nn-TransUNet consists of four parts. The first step is experimental planning and pre-processing. In this step, due to the heuristic rule, a plan for preprocessing and network training is generated, and then preprocessing is done automatically. The network training will be processed after pre-processing. In the next stage, the trained model could be used to make predictions by loading checkpoints of trainable parameters with the best performance. Finally, the predictions made by the model are used for evaluation based on multiple evaluation metrics, which will be discussed in detail in the experimental section.

### 3.1. Experiment Planning and Preprocessing

As shown in Figure 1, when the image dataset is input to the pipeline, the training data will be cropped to nonzero region and the properties of the dataset (data fingerprint) is analyzed automatically for experiment planning. based on the data fingerprint, the rule-based parameters used for preprocessing and training are generated. The preprocessing related parameters are all generated according to the data fingerprint, and the parameters for networking training are also related to device limitation (i.e., GPU RAM).

Intensity normalization and resampling strategies are related to preprocessing. Intensity normalization is affected by the modality and intensity distribution of the input image. If the modality of the image dataset is CT, the pipeline will implement a percentile clipping and project each case of the dataset to a z-score region with global foreground mean and standard deviation. If the modality is not CT, the dataset is projected to the z-score region with the mean value and standard deviation of each image.

The decisive factor of the resampling strategy is the distribution of the spacings. For the resampling strategy, third-order splines are used in-plane and nearest-neighbor out-of-plane if the spacings show an anisotropic tendency. In other cases, only third order splines are implemented. For the annotation resampling strategy, the dataset is first coded in one-hot. Then, if the dataset is anisotropic, linear interpolation is used in the plane and out-of-plane nearest neighbors are used. In other situations, only linear interpolation is implemented.

### 3.2. Network Training

#### 3.2.1. Network Architecture

**Figure 4:**
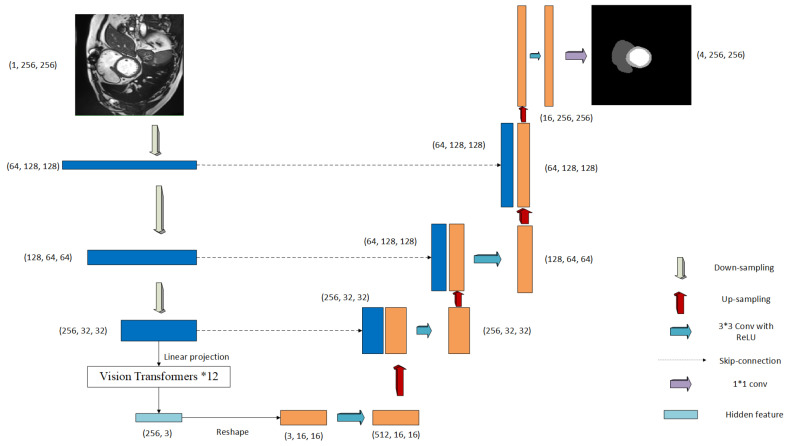
Network architecture for nn-TransUNet [20].

As the name suggests, the network used for architecture for nn-TransUNet is TransUNet. As shown in Figure 4, for this method, three layers of skip-connection are used and the number of vision transformers used is set to be 12 by default.

#### 3.2.2. Rule-Based Parameters

The most effective parameter, the image target spacing, has an impact on the other parameters that influence network training such as network topology, patch size, and batch size, determined by the distribution of dataset spacing. If the dataset is anisotropic, the image target spacing is the tenth percentile on the axis with the lowest resolution, and the median on the other axes. If the dataset is not anisotropic, the image target spacing is set to be the median spacing on each axis (the median spacing is calculated on experiment planning stage).

Since this method is designed to adapt the input dataset of multiple sizes, the topology is able to dynamically change to adapt to each input size. the size of the input dataset is defined after cropping and preprocessing based on the median resampled shape and image target spacing. Then the patch size and batch size will be defined accordingly during the training process. However, the input image size and patch size are not only defined by the size and spacing distribution of the input images, but also device limitations. In real-world training process, especially on personal computers, computational resources are limited, which means that batch sizes and patch sizes are limited, otherwise the RAM of the GPU could be exceeded and terminate the training process. With this in mind, the pipeline computes an appropriate combination of batch size and patch size, combining the median shape of the resampled dataset, image target spacing, and GPU RAM to ensure that the network can be trained and fully utilize GPU RAM.

#### 3.2.3. Fixed Parameters

In addition to the automatic process based on the heuristic rules, the pipeline also uses a fixed set of parameters. The fixed parameters consist of the settings that are empirically used for network training. These parameters have proven to have a decent performance on multiple network training. The details of the fixed parameters are presented below.

Loss Function

Essentially, a segmentation task is a classification task in deep learning. The segmentation algorithm will classify each image pixel-wise to accomplish a segmentation task. Thus, cross-entropy, a classic loss function for the classification tasks in deep learning, is always used for training and evaluation of image segmentation. Dice is an evaluation metric for image segmentation. The performance of the segmentation algorithm increases with the dice score. In this method, a combination of cross-entropy loss and dice loss is used to train the TransUNet. The format of the loss function is as follows:

(4) $$L=L_{CE}+L_{dice}$$

where $L_{CE}$ stands for cross-entropy loss, while $L_{dice}$ is the value of dice Loss. The equation of multi-classes (the number of classes larger than 2) cross-entropy loss is shown as follows:

(5) $$L_{CE}=\sum_{i=1}^{C}y_{i}log\left(p_{i}\right)$$

where *C* is the number of classes in classification task, $y_{i}$ is the binary representation (0 stands for negative, 1 for positive) if it is correct for class label *i* while $p_{i}$ is the probability that class label *i* is correct. The dice loss which is implemented in the paper is proposed by Sudre et al. [24]. The implementation of dice loss for multi-classes is shown below:

(6) $$L_{dice}=-\frac{2}{\left|K\right|}\sum_{k\in K}\frac{\sum_{i\in I}u_{i}^{k}v_{i}^{k}}{\sum_{i\in I}u_{i}^{k}+\sum_{i\in I}v_{i}^{k}}$$

where *u* is the output of TransUNet in softmax format and *v* is the one-hot encoding representation of the foreground segmentation map. i∈I is the number of pixels in the training case while k∈K is the number of classes.

Optimizer

A stochastic gradient descent (SGD) optimizer with a nestrov momentum of 0.99 is used for network training. A poly learning rate is implemented with an initial learning rate of 0.1. The learning rate is reduced by a factor of 5 per epoch until the training process is stopped.

Data augmentation

Lack of training cases is a common problem for medical image segmentation. This leads to overfitting when the amount of training data is limited. A potential solution to the problem is to increase the difficulty for network training, which could be achieved by data augmentation [25]. For the proposed method, a wide range of data augmentation methods have been implemented continuing the use of the nnU-net setup, including gamma correction of mirroring, Gaussian noise, Gaussian blur, contrast, brightness, low-resolution simulation, and rotation. The implementation of data augmentation is achieved using the BatchGenerators package which is available at github.com/MIC-DKFZ/batchgenerators.

Training procedure

In the training procedure, the total number of training epochs is fixed to 500 and each epoch includes 250 batch iterations. For each image in a batch, a random channel of images will be chosen to train the network, which means that multiple training samples can be extracted from a single training case. It can also prevent the model from overfitting, which may be caused by a lack of training cases. No early stopping criteria are used for training to ensure that the best performance in the training procedure is not missed. There is no negative effect on the training process as the best-performing checkpoints are automatically saved.

### 3.3. Predict

In training procedure, each checkpoint will be saved dynamically (the newest best checkpoint will replace the last best checkpoint when it appears) per epoch according to the value of validation loss. After the network training procedure is completed, the pipeline loads the weights from the best checkpoint for prediction.

### 3.4. Evaluation

The prediction output of the test set will be evaluated by three evaluation metrics: Dice Similarity Coefficient (DSC), Hausdorff Distance (HD), and Jaccard similarity coefficient (JSC), which are commonly used for evaluating the performance of image segmentation algorithms.

#### 3.4.1. Dice Similarity Coefficient (DSC)

The equation of Dice similarity coefficient [26] is as follows:

(7) $$DSC=\frac{2TP}{2TP+FP+FN}$$

where TP stands for true positive, FP is false positive, and FN is false negative. TP, FP, TN, and FN are terms which are used for calculating the metrics for deep learning method evaluation. In terms of image segmentation in pixel level, given the classification label *X*, TP means the classification of the pixel is correct, and the value is *X*, FP means the classification of the pixel is incorrect with the value *X*. TN means the classification of the pixel is correct, and the value is not *X* while FN stands for the classification of the pixel is incorrect with the value not *X*.

#### 3.4.2. Hausdorff Distance (HD)

Hausdorff distance [27] is a measure of the maximum distance from one group to the nearest point in another group. Hausdorff distance could be regarded as a supplement of Dice similarity coefficient in image segmentation domain. Given two sets $A=\left\{a_{1},\ldots ,a_{p}\right\}$ and $B=\left\{b_{1},\ldots ,b_{p}\right\}$, the definition of HD is shown below:

(8) $$H\left(A,B\right)=max\left\{h(A,B),h(B,A)\right\}$$

(9) $$h\left(A,B\right)=max\left\{a\in A\right\}\;min\left\{b\in B\right\}\;||a-b||$$

(10) $$h\left(B,A\right)=max\left\{b\in B\right\}\;min\left\{a\in A\right\}\;||b-a||$$

#### 3.4.3. Jaccard Similarity Coefficient (JSC)

Jaccard similarity coefficient (JSC) [28] is used to compare the similarity and difference between finite sets. The larger the JSC is, the higher the sample similarity is. Given two sets *A* and *B*, the equation of JSC is shown below:

(11) $$J\left(A,B\right)=\frac{\left|A\cap B\right|}{\left|A\cup B\right|}=\frac{\left|A\cap B\right|}{\left|A\right|+\left|B\right|-\left|A\cap B\right|}$$

### 3.5. Different Settings Related to Methods

The idea of nn-TransUNet is inspired by nnU-net and TransUNet. But when implementing the method, a series of settings are modified to adapt to the dataset and training situation.

#### 3.5.1. No Cross Validation

In nnU-net, a 5-fold cross validation is implemented to select the best check point for the model while preventing the network from overfitting. According to the fact that the number of trainable parameters for TransUNet is much more than the counterpart of U-net (TransUNet has 12 more vision transformer layers), if a 5-fold cross validation is conducted on nn-TransUNet, it will be expensively time consuming (2 times more than nnU-net on a same dataset), and several techniques to deal with training cases lacking problem are implemented(e.g. data augmentation, data loading from different channels, which will be discussed in detail in the experiment section), and the number of training cases in this paper is not too rare that a cross-validation operation is necessary. In this way, the model is trained without cross validation.

#### 3.5.2. Full Training Case Resolution

In the training process of TransUNet, Chen et al. [20] cropped the input images to reduce the consumption of computing resources. Although the TransUNet model performs well when trained with cropped images, the model trained with higher resolution images tends to perform better. In this paper, we argue that the performance of the segmentation task is more important than the time consumption, which means that the performance is more important than the time saved. Considering the situation that the segmentation method of this paper is for medical usage, the importance of the evaluation metrics (DSC, HD, and JSC) is further enhanced. In the proposed method, the full resolution training case is used, which means that a single input image used for network training is kept at its original size or processed to a similar size, which preserves the major part of the information in the raw image. The purpose of pre-processing and data augmentation is to shape the size of each input image to a fixed size to match the network topology and enhance the difficulty for training, rather than saving computational resources.

#### 3.5.3. Larger Batch Size

Compared to the TransUNet training process, the proposed method uses a larger batch size. The batch size used for training the nn-TransUNet model is automatically computed to match the RAM limitation of the GPU, potentially exploiting the computing power of the GPU. The batch size is set to the maximum until the RAM limit is reached, which causes an exception for memory out-of-bounds. The use of a large batch sizes is beneficial for deep learning algorithms in multiple domains, especially for image processing tasks. A large batch size enables the use of a large learning rate, and given that the proposed method uses a poly learning rate that decays at each epoch, it is necessary to set a large initial learning rate. When performing a stochastic gradient descent (SGD), which is used for the training of nn-TransUNnet, the training process with a larger batch size tends to have less epochs to convergence, which means the training efficiency is enhanced with large batch size if the performance of the GPU is qualified. The use of batch size also leads to a more accurate computation of the gradient. Considering the assumption of the extreme cases, which means that the batch size is the number of all the training cases, the descent of the gradient will be optimal due to the fact that the gradient is computed based on the total dataset, and all cases are considered. In this case, the randomness of SGD vanishes and it becomes gradient descent. A disadvantage of large batch size is that it takes much more time to train compared to a training plan with a smaller batch size. This problem could be partially addressed by using a GPU with higher performance, and as mentioned earlier, nn-TransUNet prefers higher performance over less consumption of computational resources and training time. Therefore, a larger batch size is chosen.

## 4. Experimental Dataset and Details

In the experimental section of this study, 3 datasets are used. Automatic Cardiac Diagnosis Challenge (ACDC) dataset is mainly used for evaluation and visualization of the proposed method. Medical Segmentation Decathlon (MSD) task02 heart dataset and Myocardial Pathology Segmentation Combining Multi-sequence CMR dataset (MyoPS 2020) are used to test the adaptability of the method to different datasets.

### 4.1. Automatic Cardiac Diagnosis Challenge (ACDC) Dataset

Automatic Cardiac Diagnosis Challenge (ACDC) Dataset which was proposed by Bernard et al. [29], was the dataset for MICCAI 2017. The cases of the ACDC dataset are acquired by scanning the organs of patients in real diagnosis. The dataset was created by the University Hospital of Dijon, France. A sufficient number of cases for deep learning training methods are covered in the ACDC dataset. The dataset consists of 300 cine-MRIs gathered from 150 patients and the cases are divided into 5 classes based on their characteristics.

#### 4.1.1. Dataset Properties

The ACDC dataset consists of 5 groups of cardiac cine-MRI with the number of 30 each from different patients: normal patients; patients with less than 40% of the left ventricle and partial myocardium tend to have abnormal contractions; patients with a left ventricle proportion below 40% and a left ventricle volume below 100 mL per square meter; patients with a left ventricle mass greater than 110 g/m² and partial myocardial segmentation tend to have segmentation thicker than 15 mm in the diastole; patients with less than 40% of the right ventricle and some parts of the heart muscle tend to have abnormal contractions. The data of each patient includes two raw MRIs, end diastolic (ED) and end systolic (ES). The segmentation task of ACDC dataset contains three classes for classification: left ventricle (LV), right ventricle (RV) and myocardium (MYO).

#### 4.1.2. Dataset Settings for the Proposed Method

Considering that the ground truth of the ACDC test set is not released, a division of the ACDC training set is made to generate a new dataset with training set and test set. The training set of the original ACDC dataset is randomly divided into a new training set, a validation set and a testing set. The training set contains 180 training cases (from 90 patients) while the testing set consists of 20 testing cases (from 10 patients) for evaluation. 36 of the training cases are chosen as the validation set.

### 4.2. Medical Segmentation Decathlon Task02 Heart DATASET (MSD02)

MSD02 was a dataset released by Antonelli et al. [30] for the heart MRI segmentation task of Medical Segmentation Decathlon. The dataset consists of 30 MRIs. The label of each case contains one class, left atrium (LA). We divided MSD02 dataset into a format of 20-5-5 for training, validation and testing.

### 4.3. Myocardial Pathology Segmentation Combining Multi-sequence CMR Dataset (MyoPS 2020)

MyoPS 2020 dataset released by Zhuang [31,32] was the heart MRI dataset for MICCAI 2020. The dataset contains 30 cases. The segmentation tasks of MyoPS 2020 dataset consist of five classes: left ventricular blood pool (LV), right ventricular blood pool (RV), LV normal myocardium (MYO), LV myocardial edema (ME), LV myocardial scars (MS). The dataset contains 25 cases, and is devided into 15 training cases, 5 validation cases and 5 testing cases.

### 4.4. Implementation Details

The experiment for nn-TransUNet is conducted in a single NVIDIA GeForce RTX 3090 GPU with 24GB of RAM. The details of the experiment setting are discussed below.

#### 4.4.1. Data Fingerprints BASED on Parameters

The experiment plan generated according to the data fingerprint analyzed by the pipeline and the fixed parameters for training process of all datasets are shown in Table 1, Table 2 and Table 3.

A small change in the patch size is implemented. For instance, the initial patch size is decided by the median shape of the dataset, which is (256, 224) for ACDC dataset. However, the TransUNet network requires the input size of each dimension to be equal. Thus, the patch size is set to be (256, 256) to adapt to the network topology.

#### 4.4.2. Ablation Study of Training Data Size

To test the performance of the model in different number of training cases, three scenarios of ACDC dataset are set to increase the difficulty of network training:

1. 144 training cases, 36 validation cases, 20 testing cases (Full dataset);
2. 72 training cases, 18 validation cases, 20 testing cases (1/2 dataset);
3. 36 training cases, 9 validation cases, 20 testing cases (1/4 dataset).

All of the training and validation cases used for the scenarios of the 1/2 dataset and 1/4 dataset are selected randomly from the full dataset. The 20 testing cases are fixed as testing for each scenario.

## 5. Results and Discussion

### 5.1. Results of Training Data Size Ablation Study

In the result section, three metrics are used to evaluate the performance of nn-TransUNet: Dice similarity coefficient (DSC) and Hausdorff distance (HD) and Jaccard similarity coefficient (JSC).

As mentioned above, three scenarios are set to test the performance of nn-TransUNet. The metric scores for the three scenarios are shown in Table 4. As shown in table, the Dice Similarity Coefficient of the model decreases with the number of training cases decreased. When the number of training cases is set to 1/4 of the full dataset, which is the minimum, the average DSC is 87.0, which is a high DSC score. As the number of training cases decreases, the performance shown by the HD and JSC scores also decreases. But the difference between the 1/2 dataset and the 1/4 dataset scenarios is significant. The performance in each scenario shows that nn-TransUNet is able to perform well in situations where the training case is lacking. However, if the number of training cases is too small, significant performance degradation can occur.

### 5.2. Quantitative Evaluation

To test the performance of the proposed method, five methods are chosen for comparison including the baseline models (i.e., TransUNet and nnU-net). The results on the selected datasets are shown in Table 5, Table 6 and Table 7. As suggested by Table 5, the average DSC score, the DSC score per class and the JSC score of nn-TransUNet rank 1st while its HD score ranked 2nd on the ACDC dataset. As shown in Table 6, the DSC, HD and JSC scores of nn-TransUNet rank 1st among all the methods on the MSD02 dataset. Table 7 shows that nn-TransUNet prevails on most of the evaluation metrics except DSC scores of Myo and MS class (both ranked 2nd) on n MyoPS 2020 dataset. The evaluation metrics shown in the above Tables strongly demonstrate that nn-TransUNet achieves state-of-the-art performance on multiple heart MRI segmentation tasks and is capable of segmentation tasks on different MRI datasets.

### 5.3. Qualitative Evaluation

**Figure 5:**
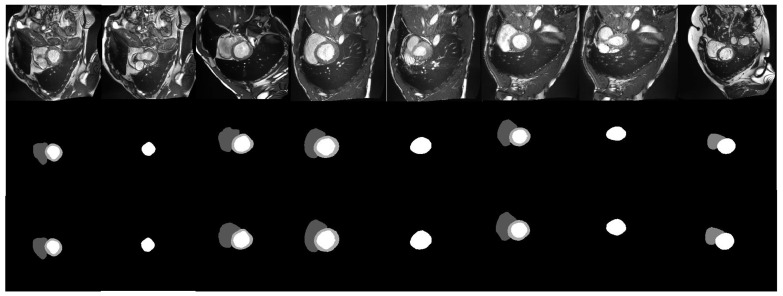
Visualization of 8 testing cases.

Eight testing cases are chosen to show the qualitative evaluation of nn-TransUNet on the ACDC dataset. A visualization of the 8 cases is shown in Figure 5. The first row is the raw images, the second row is the corresponding prediction generated by nn-TransUNet, and the third row is the ground truth images for the test case. As shown in Figure 5, the shape and volume of the predicted images match well with the ground truth images, which indicates that the performance of nn-TransUNet is good.

**Figure 6:**
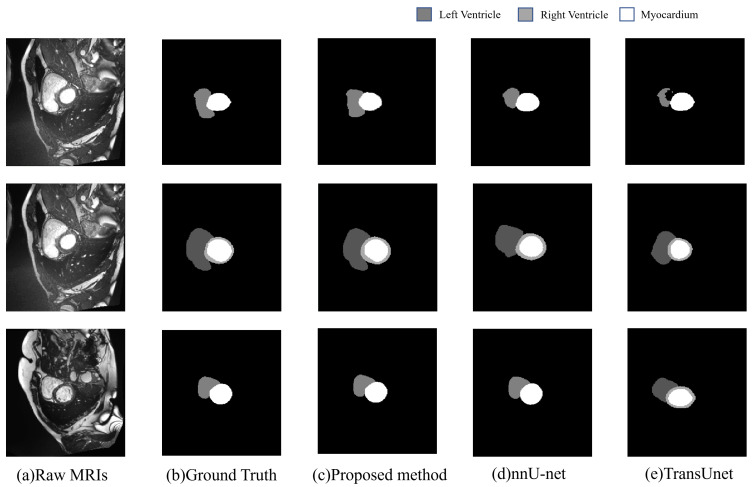
Qualitative evaluation of different methods by visualization.

As shown in Figure 6, the comparison between the proposed method and its baseline models (nnU-net and TransUNet) on ACDC dataset is presented. It can be seen in Figure 6 that the performance of nn-TransUNet is favorable compared to its baseline model. In the first and second row, the segmentation maps that nn-TransUNet generated are the most similar to the ground truth images, especially on the LV (Left Ventricle) part. It indicates that the segmentation accuracy of nn-TransUNet is better than that of nnU-net and TransUNet. In the third row, the difference between the segmentation maps of nn-TransUNet and nnU-net is not obvious except that the edges of each segment presented by nn-TransUNet tend to have more details than their counterparts presented by nnU-net. But both methods are significantly better than TransUNet, which had false positive results in its predictions. It shows that the experiment planning pipeline implemented in the proposed method can reduce the network error rate. In summary, by combining the TransUNet network with the automated experimental planning pipeline of nnUNet, nn-TransUNet prevails in the comparison to its baseline models. It also reveals that a proper training and preprocessing plan can boost the performance of a network model even when its performance is already good, which is the starting intuition of this paper.

## 6. Conclusions

In this paper, nn-TransUNet, an automatic deep learning pipeline for cardiac MRI segmentation, is proposed. nn-TransUNet is a combination of nnU-net, an automatic deep learning image segmentation training plan generative frame based on generic U-net and TransUNet, a deep learning network architecture based on vision transformers, which is an alternative choice for medical image segmentation alongside convolutional neural networks (CNNs). The nn-TransUNet uses TransUNet as the network architecture and is able to generate appropriate preprocessing and training plans based on the properties of the dataset. The evaluation metrics of the proposed model on multiple datasets achieve higher scores than several deep learning-based methods, which indicates that nn-TransUNet achieves state-of-the-art performance on heart image segmentation tasks and is capable for various heart MRI datasets. However, there are limitations. The implementation of the vision transformer increases the number of trainable parameters, which aggravates the consumption of computational resources and the increases the training time of the model compared to nnU-net. The other limitation is that the performance of the proposed models degrades sharply when the input MRIs are abnormal (missing segments of normal heart organs). In the future, we will simplify the neural network architecture to reduce the time and computational resource consumption, and balance the portions of normal heart MRIs and abnormal MRIs to increase the performance of nn-TransUNet on abnormal heart MRI cases. The proposed method can boost the research on other domains by outputting accurate segmentation maps. An implementation of nn-TransUNet for other medical image segmentation tasks, or even for other domains such as road segmentation, radiomics, 3D models of human anatomical regions or surgical planning, would be interesting and promising. We will show these implementations in a follow-up study.

## Figures and Tables

**Table 1:** Data fingerprints-based parameters.

Items	Values
Distribution of Spacings (mean)	(10, 1.25, 1.25)
Median shape	(256, 224)
Image modality	MRI
Batch size	36
Patch size	(256, 256)
Learning rate	poly learning rate with initial value 0.01
Optimizer	SGD with nestrov momentum
Epochs	500 epochs with 250 iterations per epoch

**Table 2:** MSD02 Data fingerprints-based parameters.

Items	Values
Distribution of Spacings (mean)	(1.37, 1.25, 1.25)
Median shape	(320, 232)
Image modality	MRI
Batch size	40
Patch size	(320, 320)
Learning rate	poly learning rate with initial value 0.01
Optimizer	SGD with nestrov momentum
Epochs	500 epochs with 250 iterations per epoch

**Table 3:** MyoPS 2020 Data fingerprints-based parameters.

Items	Values
Distribution of Spacings (mean)	(14, 0.73, 0.73)
Median shape	(512, 512)
Image modality	MRI
Batch size	4
Patch size	(512, 512)
Learning rate	poly learning rate with initial value 0.01
Optimizer	SGD with nestrov momentum
Epochs	500 epochs with 250 iterations per epoch

**Table 4:** Results in different training scenarios on ACDC dataset (average DSC (%), DSC (%) of each class (center ventricle, right ventricle, myocardium), average HD (mm)).

Scenario	Avg.DSC↑	LV↑	RV↑	Myo↑	HD↓	JSC↑
Full dataset	93.6	96.3	93.1	91.5	9.1	88.3
1/2 dataset	89.6	94.0	88.5	86.3	10.4	82.5
1/4 dataset	87.0	90.3	86.7	84.1	11.5	78.7

**Table 5:** Results on ACDC dataset (average DSC (%), DSC (%) of each class (center ventricle, right ventricle, myocardium), average HD (mm), average JSC (%)).

Scenario	Avg.DSC↑	LV↑	RV↑	Myo↑	HD↓	JSC↑
R50 UNet [10]	87.6	88.1	91.0	83.7	11.4	81.8
R50 Att-UNet [33]	86.7	87.5	91.8	80.9	13.4	78.8
ViT [17]	81.3	80.3	92.1	71.5	18.3	69.6
nnU-net [15]	90.9	91.0	92.3	89.5	8.7	85.8
TransUNet [20]	89.7	86.3	95.6	87.3	12.0	82.5
Proposed method	93.6	96.3	93.1	91.5	9.1	88.3

**Table 6:** Results on MSD02 dataset (DSC (%), average HD (mm), average JSC (%)).

Scenario	DSC↑	HD↓	JSC↑
R50 UNet [10]	84.4	4.0	76.6
R50 Att-UNet [33]	85.9	4.3	83.3
ViT [17]	80.3	5.8	65.5
nnU-net [15]	92.5	3.1	86.1
TransUNet [20]	88.4	3.9	81.6
Proposed method	93.9	2.8	88.6

**Table 7:** Results on MyoPS 2020 dataset (average DSC (%), DSC (%) of each class (center ventricular blood pool, right ventricular blood pool, LV normal myocardium, LV myocardial edema, LV myocardial scars), average HD (mm), average JSC (%)).

Scenario	Avg.DSC↑	LV↑	RV↑	Myo↑	ME↑	MS↑	HD↓	JSC↑
R50 UNet [10]	69.0	87.5	90.7	78.3	34.7	53.7	12.5	56.5
R50 Att-UNet [33]	71.6	89.3	89.9	73.0	50.3	55.6	13.9	50.4
ViT [17]	63.7	88.8	90.5	66.4	29.2	43.7	15.1	40.9
nnU-net [15]	73.8	93.3	95.3	71.6	36.6	72.3	10.8	55.8
TransUNet [20]	74.0	92.0	90.1	81.9	40.0	66.3	7.8	58.5
Proposed method	78.7	94.5	94.1	81.1	54.8	69.0	5.2	67.4

## Data Availability

The heart MRI datasets are available at www.creatis.insa-lyon.fr/Challenge/acdc, medicaldecathlon.com/ and www.sdspeople.fudan.edu.cn/zhuangxiahai/0/myops20/index.html accessed on 1 August 2022.

## References

[1] Anderson K.M., Odell P.M., Wilson P.W., Kannel W.B. (1991). Cardiovascular disease risk profiles. Am. Heart J..

[2] Roacho-Pérez J.A., Garza-Treviño E.N., Moncada-Saucedo N.K., Carriquiry-Chequer P.A., Valencia-Gómez L.E., Matthews E.R., Gómez-Flores V., Simental-Mendía M., Delgado-Gonzalez P., Delgado-Gallegos J.L. (2022). Artificial Scaffolds in Cardiac Tissue Engineering. Life.

[3] Timpau A.S., Miftode R.S., Leca D., Timpau R., Miftode I.L., Petris A.O., Costache I.I., Mitu O., Nicolae A., Oancea A. (2022). A real Pandora’s box in in Pandemic Times: A Narrative Review on the Acute Cardiac Injury Due to COVID-19. Life.

[4] Bhattacharya S., Reddy Maddikunta P.K., Pham Q.V., Gadekallu T.R., Krishnan S S.R., Chowdhary C.L., Alazab M., Jalil Piran M. (2021). Deep learning and medical image processing for coronavirus (COVID-19) pandemic: A survey. Sustain. Cities Soc..

[5] Ozturk T., Talo M., Yildirim E.A., Baloglu U.B., Yildirim O., Rajendra Acharya U. (2020). Automated detection of COVID-19 cases using deep neural networks with X-ray images. Comput. Biol. Med..

[6] Zhou Y., Yang Z., Guo Y., Geng S., Gao S., Ye S., Hu Y., Wang Y. (2020). A New Predictor of Disease Severity in Patients with COVID-19 in Wuhan, China. medRxiv.

[7] Gozes O., Frid-Adar M., Greenspan H., Browning P.D., Zhang H., Ji W., Bernheim A., Siegel E. (2020). Rapid AI Development Cycle for the Coronavirus (COVID-19) Pandemic: Initial Results for Automated Detection & Patient Monitoring using Deep Learning CT Image Analysis. arXiv.

[8] Jin X., Jiang Q., Chu X., Lang X., Yao S., Li K., Zhou W. (2020). Brain Medical Image Fusion Using L2-Norm-Based Features and Fuzzy-Weighted Measurements in 2-D Littlewood–Paley EWT Domain. IEEE Trans. Instrum. Meas..

[9] Long J., Shelhamer E., Darrell T. Fully Convolutional Networks for Semantic Segmentation. Proceedings of the IEEE Conference on Computer Vision and Pattern Recognition (CVPR).

[10] Ronneberger O., Fischer P., Brox T., Navab N., Hornegger J., Wells W.M., Frangi A.F. (2015). U-Net: Convolutional Networks for Biomedical Image Segmentation. Proceedings of the Medical Image Computing and Computer-Assisted Intervention—MICCAI 2015.

[11] Milletari F., Navab N., Ahmadi S.A. V-Net: Fully Convolutional Neural Networks for Volumetric Medical Image Segmentation. Proceedings of the 2016 Fourth International Conference on 3D Vision (3DV).

[12] Zhou Z., Rahman Siddiquee M.M., Tajbakhsh N., Liang J., Stoyanov D., Taylor Z., Carneiro G., Syeda-Mahmood T., Martel A., Maier-Hein L., Tavares J.M.R., Bradley A., Papa J.P., Belagiannis V. (2018). UNet++: A Nested U-Net Architecture for Medical Image Segmentation. Proceedings of the Deep Learning in Medical Image Analysis and Multimodal Learning for Clinical Decision Support.

[13] Mehta S., Mercan E., Bartlett J., Weaver D., Elmore J.G., Shapiro L., Frangi A.F., Schnabel J.A., Davatzikos C., Alberola-López C., Fichtinger G. (2018). Y-Net: Joint Segmentation and Classification for Diagnosis of Breast Biopsy Images. Proceedings of the Medical Image Computing and Computer Assisted Intervention—MICCAI 2018.

[14] Mehta R., Sivaswamy J. M-net: A Convolutional Neural Network for deep brain structure segmentation. Proceedings of the 2017 IEEE 14th International Symposium on Biomedical Imaging (ISBI 2017).

[15] Isensee F., Jaeger P.F., Kohl S.A., Petersen J., Maier-Hein K.H. (2021). nnU-Net: A self-configuring method for deep learning-based biomedical image segmentation. Nat. Methods.

[16] Vaswani A., Shazeer N., Parmar N., Uszkoreit J., Jones L., Gomez A.N., Kaiser L.u., Polosukhin I., Guyon I., Luxburg U.V., Bengio S., Wallach H., Fergus R., Vishwanathan S., Garnett R. (2017). Attention is All you Need. Proceedings of the Advances in Neural Information Processing Systems.

[17] Dosovitskiy A., Beyer L., Kolesnikov A., Weissenborn D., Zhai X., Unterthiner T., Dehghani M., Minderer M., Heigold G., Gelly S. (2020). An Image is Worth 16x16 Words: Transformers for Image Recognition at Scale. arXiv.

[18] Han K., Wang Y., Chen H., Chen X., Guo J., Liu Z., Tang Y., Xiao A., Xu C., Xu Y. (2020). A Survey on Visual Transformer. arXiv.

[19] Tragakis A., Kaul C., Murray-Smith R., Husmeier D. (2022). The Fully Convolutional Transformer for Medical Image Segmentation. arXiv.

[20] Chen J., Lu Y., Yu Q., Luo X., Adeli E., Wang Y., Lu L., Yuille A.L., Zhou Y. (2021). TransUNet: Transformers Make Strong Encoders for Medical Image Segmentation. arXiv.

[21] Shaw P., Uszkoreit J., Vaswani A. (2018). Self-Attention with Relative Position Representations. arXiv.

[22] Wang E., Yu Q., Chen Y., Slamu W., Luo X. (2022). Multi-modal knowledge graphs representation learning via multi-headed self-attention. Inf. Fusion.

[23] Riedmiller M., Lernen A. (2014). Multi layer perceptron. Machine Learning Lab Special Lecture.

[24] Sudre C.H., Li W., Vercauteren T., Ourselin S., Jorge Cardoso M., Cardoso M.J., Arbel T., Carneiro G., Syeda-Mahmood T., Tavares J.M.R., Moradi M., Bradley A., Greenspan H., Papa J.P., Madabhushi A. (2017). Generalised Dice Overlap as a Deep Learning Loss Function for Highly Unbalanced Segmentations. Proceedings of the Deep Learning in Medical Image Analysis and Multimodal Learning for Clinical Decision Support.

[25] Perez L., Wang J. (2017). The Effectiveness of Data Augmentation in Image Classification using Deep Learning. arXiv.

[26] Jimenez S., Gonzalez F.A., Gelbukh A. (2016). Mathematical properties of soft cardinality: Enhancing Jaccard, Dice and cosine similarity measures with element-wise distance. Inf. Sci..

[27] Huttenlocher D., Klanderman G., Rucklidge W. (1993). Comparing images using the Hausdorff distance. IEEE Trans. Pattern Anal. Mach. Intell..

[28] Eelbode T., Bertels J., Berman M., Vandermeulen D., Maes F., Bisschops R., Blaschko M.B. (2020). Optimization for Medical Image Segmentation: Theory and Practice When Evaluating With Dice Score or Jaccard Index. IEEE Trans. Med. Imaging.

[29] Bernard O., Lalande A., Zotti C., Cervenansky F., Yang X., Heng P.A., Cetin I., Lekadir K., Camara O., Gonzalez Ballester M.A. (2018). Deep Learning Techniques for Automatic MRI Cardiac Multi-Structures Segmentation and Diagnosis: Is the Problem Solved?. IEEE Trans. Med. Imaging.

[30] Antonelli M., Reinke A., Bakas S., Farahani K., Kopp-Schneider A., Landman B.A., Litjens G., Menze B., Ronneberger O., Summers R.M. (2022). The medical segmentation decathlon. Nat. Commun..

[31] Zhuang X. (2019). Multivariate Mixture Model for Myocardial Segmentation Combining Multi-Source Images. IEEE Trans. Pattern Anal. Mach. Intell..

[32] Zhuang X., Ourselin S., Joskowicz L., Sabuncu M.R., Unal G., Wells W. (2016). Multivariate Mixture Model for Cardiac Segmentation from Multi-Sequence MRI. Proceedings of the Medical Image Computing and Computer-Assisted Intervention—MICCAI 2016.

[33] Schlemper J., Oktay O., Schaap M., Heinrich M., Kainz B., Glocker B., Rueckert D. (2019). Attention gated networks: Learning to leverage salient regions in medical images. Med. Image Anal..

